# Heart rate variability as a predictor of stroke course, functional outcome, and medical complications: A systematic review

**DOI:** 10.3389/fphys.2023.1115164

**Published:** 2023-02-09

**Authors:** Joanna Aftyka, Jacek Staszewski, Aleksander Dębiec, Aleksandra Pogoda-Wesołowska, Jan Żebrowski

**Affiliations:** ^1^ Faculty of Physics, Warsaw University of Technology, Warsaw, Poland; ^2^ Clinic of Neurology, Military Institute of Medicine, Warsaw, Poland

**Keywords:** heart rate variability, stoke course, acute phase, functional outcome, medical complications, systematic review, linear analysis, non-linear analysis

## Abstract

**Background:** Heart rate variability (HRV) is a non-invasive marker of autonomic nervous system function that is based on the analysis of length differences between subsequent RR intervals of the electrocardiogram. The aim of this systematic review was to assess the current knowledge gap in the utility of HRV parameters and their value as predictors of the acute stroke course.

**Methods:** A systematic review was performed in accordance with the PRISMA guidelines. Relevant articles published between 1 January 2016 and 1 November 2022 available in the PubMed, Web of Science, Scopus, and Cochrane Library databases were obtained using a systematic search strategy. The following keywords were used to screen the publications: “heart rate variability” AND/OR “HRV” AND “stroke.” The eligibility criteria that clearly identified and described outcomes and outlined restrictions on HRV measurement were pre-established by the authors. Articles assessing the relationship between HRV measured in the acute phase of stroke and at least one stroke outcome were considered. The observation period did not exceed 12 months. Studies that included patients with medical conditions influencing HRV with no established stroke etiology and non-human subjects were excluded from the analysis. To minimize the risk of bias, disagreements throughout the search and analysis were resolved by two independent supervisors.

**Results:** Of the 1,305 records obtained from the systematic search based on keywords, 36 were included in the final review. These publications provided insight into the usability of linear and non-linear HRV analysis in predicting the course, complications, and mortality of stroke. Furthermore, some modern techniques, such as HRV biofeedback, for the improvement of cognition performance after a stroke are discussed.

**Discussion:** The present study showed that HRV could be considered a promising biomarker of a stroke outcome and its complications. However, further research is needed to establish a methodology for appropriate quantification and interpretation of HRV-derived parameters.

## 1 Introduction

Stroke is classically characterized as a sudden neurological deficit attributed to acute focal injury of the central nervous system by a vascular cause, including ischemic or hemorrhagic stroke which can be further subdivided into intracerebral hemorrhage (ICH), and subarachnoid hemorrhage (SAH) (Sacco et al., 2013). Acute ischemic stroke (AIS) is responsible for more than 80% of all strokes and is related to the stenosis or occlusion of both large and small vessels attributable to thrombosis, embolization or critical hypoperfusion. AIS is the second most common cause of death and disability in adults and has a significant impact on global health expenditure (GBD 2016 Neurology Collaborators, 2019). Despite the introduction of effective recanalizing therapies for AIS and more effective management of hemorrhagic strokes, most stroke survivors require long-term rehabilitation and ongoing management. Consequences of stroke, particularly cardiac complications (e.g., rhythm disturbances or myocardial ischemia even in patients with no history of primary heart disease), pneumonia, early recurrent strokes, or secondary ICHs are frequent, and they have a significant and poor impact on short- and long-term patient outcomes (Dogan et al., 2004). Studies have revealed that within the first month following ischemic stroke onset 2%–6% of patients die from cardiac causes: acute myocardial infarction, heart failure, ventricular tachycardia/fibrillation, and cardiac arrest (Adams et al., 2003; Prosser et al., 2007). The 30-day mortality in patients with non-traumatic ICH is even higher than that in AIS, reaching approximately 40%, while only 12%–39% of surviving patients are functionally independent post-stroke.

Medical post stroke complications present potential barriers to optimal recovery, and their prevention and prediction basing on widely available biomarkers are therefore important. While the search for different biochemical or radiological predictors of the course of stroke continues, the interpretation of most biochemical markers is confounded by their covert rise, different lesion sizes in neuroimaging, and heterogeneity of stroke etiologies, limiting their application for early diagnosis and outcome prediction (Maas and Furie, 2009). Therefore, the evaluation of the predictive value of autonomic disturbances related to stroke could become an important measure for identifying high-risk populations among patients with acute stroke (Li et al., 2021).

Stroke impairs autonomic function and is associated with a predominance of sympathetic activity, which leads to an impairment in cardiovascular responses and these may impact on the stroke course and patient outcomes (De Raedt et al., 2015). However, the direct mechanisms by which cerebrovascular events may cause heart dysfunction, including changes frequently noted in an electrocardiogram (ECG), have not been clearly defined. Some reports suggest that sustained sympathetic stimulation results in structural damage to the myocardium or disturbed autonomic cardiovascular control (Togha et al., 2013).

Heart rate variability (HRV) refers to various methods used to measure the autonomic nervous system balance of its sympathetic and parasympathetic branches, and it characterizes the pattern of spontaneous heart rate fluctuations. It reflects oscillations in the interval between R-R intervals, resulting mainly from the dynamic interaction between sympathetic and parasympathetic inputs to the heart through the sinoatrial node (Malik, 1996). Some prospective studies have shown an association between HRV and stroke incidence, demonstrating that HRV was reduced in patients with AIS compared to healthy controls, and revealed that patients with stroke showing reduced HRV had increased mortality and poor functional outcome (Mäkikallio et al., 2004; Kubota et al., 2017; Lees et al., 2018). However, these reports were based on small studies with heterogeneous designs (retrospective or prospective), different follow-up period, severity of stroke and stroke etiology (ischemic or hemorrhagic), limiting the generalizability of the results. Additionally, there is no universal recommendation which HRV parameters are best for examining the autonomic nervous system. Although researchers have been traditionally using linear time-domain or frequency-domain parameters (Sethi et al., 2016; Chidambaram et al., 2017), more recently, researchers have used non-linear parameters such as approximate entropy, multiscale entropy, detrended fluctuations, and fractal dimensions (FDs) (Tobaldini et al., 2019a; Tobaldini et al., 2019b; He et al., 2019) to provide additional or alternative insights into the investigated physiological states. Furthermore, the exact mechanism responsible for poor outcomes in patients with stroke showing reduced HRV remains unclear. Reduced HRV may simply indicate a pre-existing stroke risk factor; however, brain injury itself may induce dysregulation of the autonomic network, which may subsequently influence stroke outcomes (Scheitz et al., 2018). In-depth research of these changes may help to develop novel monitoring strategies, identify high-risk populations of unfavorable outcome and novel therapeutic targets in both ischemic or hemorrhagic stroke.

A review of the current literature examining the relationship between HRV as a predictor of the course of stroke, functional outcome, and medical complications in patients with stroke indicates a knowledge gap. To the best of our knowledge, the last systematic review that met the criteria for this manuscript was published in 2018 by Lees et al. (2018). Therefore, we decided to write a new systematic review summarizing current research, because the review by Lees et al. (2018) included articles published until 2017 and did not include newer studies. The last systematic review consisted of research assessing HRV in both acute and long-term post-acute stroke periods with different follow-up times (up to several years after a stroke), which might significantly influence the results. We decided to include patients with ischemic or hemorrhagic stroke taking into account that majority of studies reporting HRV in acute cerebrovascular accidents focused mainly on AIS and, the presence and character of HRV changes and its significance in hemorrhagic strokes remain underexplored (Szabo et al., 2018). Thus, the present systematic review addresses this knowledge gap and aims to examine and discuss the most recent literature that has investigated the relationship between different HRV parameters evaluated in the acute phase of stroke with stroke complications, functional outcomes, and mortality.

## 2 Methods

This systematic review illustrates the qualitative assessment of recent HRV research as a predictor of the stroke course, functional outcomes, and medical complications. The review process was conducted according to the PRISMA Statement to systematically analyze studies on the relationship between HRV and the course of stroke (Page et al., 2021).

### 2.1 Data sources and search strategy

Relevant articles were obtained by searching the PubMed, Web of Science, Scopus, and Cochrane Library databases for studies published between 1 January 2016, and 1 November 2022. A search by “all fields” was used in PubMed. The keywords [(“heart rate variability” OR HRV) AND stroke] were used in the query. A similar search was performed for “title-abstract-keywords” in Scopus and Cochrane Library databases. In the Web of Science, a search was conducted in the “Topic” field, which corresponds to “title-abstract-keywords.”

PubMed query: *Search:* ((*“heart rate variability”*) *OR (HRV)*) *AND (stroke) Filters: English, from 2016–2022 Sort by: Publication Date* ((*“heart rate variability” [All Fields] OR “HRV” [All Fields]*) *AND* (*“stroke” [MeSH Terms] OR “stroke” [All Fields] OR “strokes” [All Fields] OR “stroke s” [All Fields]*)) *AND* ((*english [Filter]*) *AND* (*2016: 2022 [pdat]*)) *Translations stroke: “stroke” [MeSH Terms] OR “stroke” [All Fields] OR “strokes” [All Fields] OR “stroke’s” [All Fields]*.

Web of Science query: *“heart rate variability” or HRV (Topic) and stroke (Topic) and English (Languages) and Article (Document Types)|Timespan: 2016-01-01 to 2022-11-01 (Index Date*).

Scopus query: *[TITLE-ABS-KEY (“heart rate variability”) OR TITLE-ABS-KEY (hrv) AND TITLE-ABS-KEY (stroke)] AND PUBYEAR >2015 AND [LIMIT-TO (DOCTYPE, “ar”)] AND [LIMIT-TO (LANGUAGE, “English”)]*.

Cochrane query: “*heart rate variability” OR HRV in Title Abstract Keyword AND stroke in Title Abstract Keyword; Custom Range from 2016.*

During the search, in addition to narrowing down the results from 1 January 2016, to 1 November 2022, a filter was used for the language of the publication (English) and the type of publication (articles).

### 2.2 Inclusion and exclusion criteria

The PICOS model was used to define inclusion criteria (Liberati et al., 2009; O'Connor et al., 2008). Population: “patients in the acute phase of stroke”; Intervention: HRV examination; Comparators: intragroup/between group/to control group (time limited to 1 year); Outcome: mortality/functional outcome/stroke complications; Study design: cohort study, cross-sectional study, randomized controlled trial, retrospective study, prospective observational study, prospective registration study, and randomized sham-controlled pilot study.

The inclusion criteria were as follows:

- Participants/patients were in the acute phase of stroke.
- HRV was evaluated in the acute phase (<7 days after onset). All the HRV measurement methods were considered.
- The results were compared within groups, between groups, or with a control group. An observation period of <12 months was considered.
- The study provided information on at least one stroke outcome: mortality and/or functional status, stroke recurrence, and stroke complications (autonomic dysfunction, myocardial infarction, depression, cognitive impairment, or infection).
- The following study designs were analyzed: cohort, cross-sectional, randomized controlled, retrospective, prospective observational, prospective registration, and randomized sham-controlled pilot studies.

The exclusion criteria were as follows:

- Non-human subject research.
- Unknown stroke etiology (hemorrhagic/ischemic).
- Studies that included participants with medical conditions that could potentially influence HRV assessment (e.g., pacemaker rhythm and sepsis).

### 2.3 Selection of studies

Two review authors independently selected the trials for inclusion and evaluated their methodological quality. The titles and abstracts of all articles were screened to reduce the risk of bias and ambiguity. Only full peer-reviewed and English-language publications were included. Subsequently, reading the full manuscript allowed further selection. The eligibility criteria that clearly identified and described the outcomes and outlined restrictions on HRV measurements were pre-established. Two authors (JA and A.PW.) independently conducted an electronic database search and selected the included studies according to the aforementioned criteria. Duplicate records were removed and the authors (J.A and A.PW) examined the full texts to confirm the suitability of the studies. The minimum number of patients enrolled in the study was not determined. During the whole process, disagreements were resolved by consulting two independent supervisors (J.Ż and J.S).

### 2.4 Data extraction

Using the PICOS approach, two authors (J.A and A.PW) independently extracted the following data: study source (authors and year of publication), study population (sample size, sex, mean age), objective, time from stroke onset to HRV measurement, HRV analysis method, HRV parameters, endpoint, follow-up, and study design type.

### 2.5 Quality assessment and risk of bias

The Newcastle-Ottawa Scale metric (Wells et al., 2014) was used to assess methodological quality. Each included study was judged on three broad perspectives, as recommended by the Cochrane Non-Randomized Studies Methods Working Group Version 5.1.0: selection of the study groups, comparability of the groups, and ascertainment of the outcome of interest. The selection section was scored from 0 to 4 points, the comparability section from 0 to 2 points, and the outcome section from 0 to 3 points. In total, the articles received 0–9 points. Manuscripts with 7–9, 4–6, and 0–3 points were considered high quality, medium quality, and low quality, respectively. The risk of bias was independently assessed by two authors (J.A and A.PW). Any disagreements on assessments were resolved by consulting a third review author (J.S.) to arbitrate as needed.

## 3 Results

### 3.1 Study selection

**FIGURE 1:**
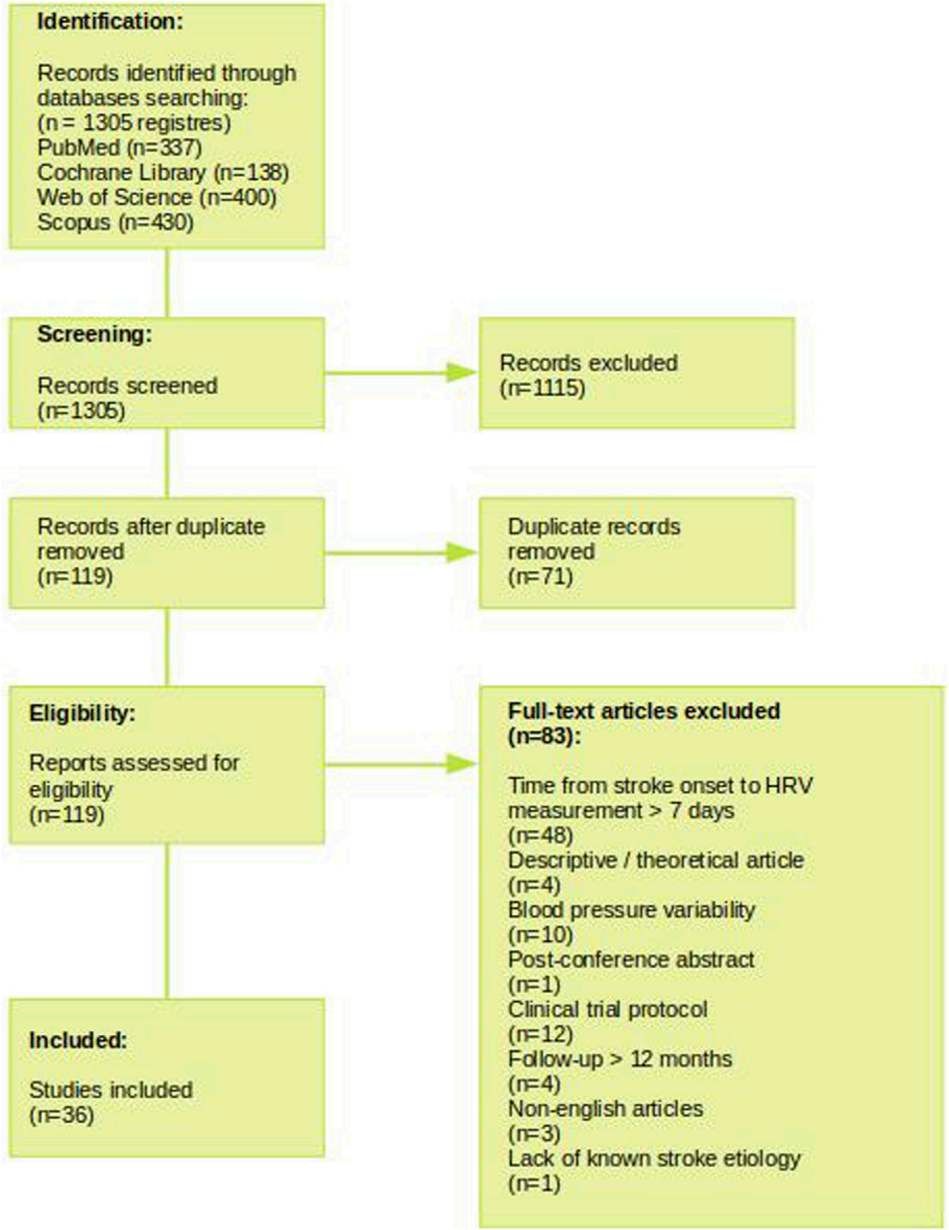
Flow chart for study search and selection methods according to the PRISMA method.

The initial search revealed 1,305 potentially interesting reviews (PubMed, 337; Cochrane Library, 138; Web of Science, 400; and Scopus, 430). These articles (titles and abstracts) were screened according to inclusion and exclusion criteria. The first assessment of the eligibility of the articles and compliance with the inclusion criteria based on the title and abstract excluded 1,115 articles. Duplicate records were removed (n = 71), and the full texts were examined to confirm the suitability of the studies. Of the 1,305 articles, full text of 119 articles were read by the researchers, which were then assessed against the inclusion criteria, resulting in exclusion of 83 articles. Finally, 36 publications were included in the analysis, comprising of 29 studies on ischemic and 8 studies on hemorrhagic stroke. Figure 1 shows the PRISMA flow diagram for selecting articles included in this manuscript.

### 3.2 Study characteristics

Supplementary Table S1 summarizes the 36 studies included in this manuscript. Study quality analysis using the Newcastle-Ottawa Scale showed that the majority of studies were of medium quality (31/36; 86%) and none were of low quality (Supplementary Table S2).

#### 3.2.1 Post-stroke outcomes

##### 3.2.1.1 Linear HRV analysis

Linear HRV analysis has been used in most studies. In 2016, Sethi et al. (2016) analyzed HRV in 13 patients with AIS who underwent a 24-h Holter ECG within 3 days of admission to a rehabilitation clinic. The level of motor impairment was assessed in all patients 3 months after stroke onset. The results demonstrated a strong positive and significant correlation between HRV and impairment of the affected upper (r = 0.70, *p* = 0.01) and lower extremities (r = 0.60, *p* = 0.03). This relationship was particularly pronounced in patients with severe initial hemiparesis (upper extremity: r = 0.61, *p* = 0.04; lower extremity: r = 0.70, *p* = 0.04). The authors concluded that HRV was strongly associated with motor outcomes after AIS and was a promising marker for investigating the mechanisms involved in motor regeneration following stroke.

Xu et al. (2016) in their paper investigated the autonomic function of the heart in patients with AIS by calculating the acceleration and deceleration capacities of the heart rate. Using linear HRV time-domain analysis methods [standard deviation of normal-to-normal RR interval (SDNN) and the square root of the mean of the sum of the squares of differences between adjacent NN intervals (RMSSD)], they showed that in patients with hemispherical infarction, acceleration and deceleration capacities decreased as a result of both vagal and sympathetic nerve modulation. Correlation analysis revealed that deceleration capacity, acceleration capacity, and SDNN were negatively correlated with stroke severity, as assessed by National Institutes of Health Stroke Scale (NIHSS) scores (r = −0.279, r = −0.266, and r = −0.319; *p* = 0.027, *p* = 0.035, and *p* = 0.011, respectively).

Another study by Candemir and Onder (2020) on 148 patients with AIS and 80 controls who had at least 20 h of Holter ECGs performed revealed that all HRV parameters including time-domain (SDSD, SDNN, SDANN, RMSSD, pNN50) and frequency-domain parameters of HRV (very-low frequency (VLF), low frequency (LF), high frequency (HF), LF/HF ratio) were significantly reduced in the stroke group. Furthermore, the study aimed to determine whether heart rate turbulence (HRT) differed in patients with AIS as compared to that in the control group. The two phases of HRT were quantified numerically: turbulence onset (TO) and turbulence slope (TS). TO was defined as the percentage change in RR intervals after ventricular premature beats compared to the pre-ventricular premature beats period. TS was defined as the maximum positive regression slope obtained over any five consecutive sinus RR intervals within the first 15 sinus RR intervals following ventricular premature beats. TO < 0 (negative TO) and TS > 2.5 ms/RR (positive TS) were considered normal values. The results demonstrated that the TO and TS values differed significantly between the control and stroke groups (*p* < 0.001 and *p* = 0.005, respectively). Both HRT parameters (TO and TS) were normal in 41.9% (31/74) of the patients with left cerebral infarct and in only 24.3% (18/74) of the patients with right cerebral infarct. In addition, either of the two HRT parameters (TO or TS) were disrupted in 33.8% (25) of the patients with left cerebral infarction and in 52.7% (39) of the patients with right cerebral infarction (*p* = 0.020).

An important study was published by Zhang et al. (2021) who studied 106 patients with emergent large vessel occlusion treated with mechanical thrombectomy under general anesthesia. Heart rate and systolic and diastolic blood pressure (BP) were recorded during mechanical thrombectomy. In this analysis, it was found that a decreased LF/HF ratio was correlated with an unfavorable functional outcome (modified Rankin Scale [mRS] 2 (odds ratio [OR], 0.65; 95% confidence interval [CI], 0.157–0.839; *p* = 0.018) and dependence (mRS 3 (OR, 0.7; 95% CI, 0.360–0.914; *p* = 0.02) at 3 months. The authors also revealed that emergent large vessel occlusion in the right anterior circulation correlated with a lower LF/HF ratio than that on the contralateral side (*p* < 0.05).

Brunetti et al. (2019) showed that autonomic dysfunction in AIS is greater in patients with right-sided stroke (n = 21) than in those with left-sided stroke (n = 21) and closely depends on the wake stage of sleep. Observations were based on overnight polysomnographic examination.

Another study by Zhao et al. (2020) analyzed the HRV records of 186 patients followed for 1 year with regard to the localization and the severity of the stroke. HRV parameters (SDNN, mean RR, PMSSD, pNN50) in the LAA (large artery atherosclerotic infarction) group were significantly lower compared to that in the other stroke location. A significantly lower HRV occurred in the patients who experienced unfavorable outcome (mRS 3–5) and had moderate disability (NIHSS 5–14); lower SDNN was independently associated with unfavorable outcome and higher NIHSS at discharge. Only SDNN showed predictive value for mRS≥3 after 1 year.

Li et al. (2021) performed 24-h Holter ECG in patients diagnosed with ischemic stroke or transient ischemic attack (TIA) and assessed SDNN and RMSSD. They proved that the rate of neurological disability 90 days after stroke and the recurrence of stroke tended to decrease with a gradual increase in SDNN. No clear relationship was found between SDNN, death from cardiovascular causes, RMSSD, 90-day neurological disability, recurrent stroke, or cardiovascular death.

Castro et al. (2022) examined the relationship between HRV and baroreflex sensitivity (BRS) in hyperacute ischemic stroke (several hours after stroke onset) and its impact on clinical and radiological outcomes. Researchers assessed outcomes at discharge and 7 and 90 days after stroke. In their work, they used both linear analysis methods: linear (SDNN, LF, and HF) and non-linear methods (Sample Entropy, Fuzzy Entropy). They revealed that the higher the high-frequency power of the BRS and HRV, the higher the gain of cerebral autoregulation (*p* < 0.05). In addition, increased vagal modulation correlated with a greater ischemic area (*p* < 0.05) but not with the clinical outcome.

Wei et al. (2017) investigated the relationship between autonomic function and stroke in a group of patients with renal dysfunction. Kidney damage was assessed using estimated glomerular filtration rate (eGFR), and stroke severity was rated according to the NIHSS. The published results indicate that autonomic dysfunction increases with the progression of eGFR stage in patients with AIS. In patients with mild or moderately decreased eGFR, the measures of SDANN, VLF, LF, and LH/HF were significantly lower than in patients with normal eGFR (*p* = 0.022 (for SDANN), 0.043 (for VLF), 0.023 (for LF), and 0.001 (for LF and HF)).

Tian et al. (2019) performed the spectral analysis of HF, LF, and VLF and analyzed the association between HRV and autonomic dysregulation. They indicated that the LF and LF/HF ratio of the BP variability were positively correlated with parasympathetic parameters, while the VLF of HRV was negatively correlated with sympathetic parameters, and the others were positively correlated with parasympathetic parameters.

In another important study by Szabo et al. (2018), the patients in the acute phase of ICH showed significantly lower HF (*p* = 0.01), LF (*p* < 0.001), and LF/HF (*p* < 0.001) and significantly higher normalized HF (*p* = 0.03) than those in the control group. These findings were probably related to abnormal sympathetic and parasympathetic responses. The authors speculated that parasympathetic shift resulted from sudden increase in intracranial pressure in ICH patients and concluded that in the acute phase of ICH, autonomic changes are visible and are associated with a poor clinical prognosis.

Tsai et al. (2019) analyzed differences in BRS depending on the subtypes of AIS. Patients with AIS had a higher LF/HF ratio than those in the control group (*p* < 0.05). They also indicated that BRS was significantly reduced (*p* < 0.01) in patients with a major hemispheric infarction or a brainstem infarction compared to those with a small deep hemispheric infarction.

Tang et al. (2020) indicated that patients with a low LF/HF ratio determined within the first 7 days of stroke will have worse results according to the NIHSS scale in the longer term (3 months).

Miwa et al. (2021) analyzed the impact of heart rate and HRV on poorer clinical outcomes. They showed that higher mean heart rates and average real variability over the first 24 h were associated with poor prognosis in acute ICH. The average real HRV also correlated with hematoma expansion after 24 h.

Wang et al. (2022) described cardiovascular autonomic modulation assessment in patients with posterior circulation ischemic stroke with and without brainstem involvement. The results indicate that in the acute phase of stroke, patients with posterior circulation ischemic stroke concomitant with occipital, thalamic, cerebellar, or brainstem infarction had lower cardiovagal modulation, compromised baroreflex, and increased peripheral sympathetic modulation, whereas after occipital stroke, the advantage of the sympathetic system was greater (LF/HF ratio).

##### 3.2.1.2 Non-linear HRV analysis

In in Supplementary Table S3 association between HRV and AIS or ICH evaluated by non-linear analysis is presented. This has been published by Tobaldini et al. (2019a); Tobaldini et al. (2019b) and Chen et al., 2018 and Sykora et al., 2020. A novel HRV analysis with the use of symbolic analysis consists of three formulas: the pattern with no variation (0V%, representing the sympathetic modulation), the pattern with two similar variations, and the pattern with two different variations (2LV% and 2UV% as markers of vagal modulation, respectively); this has been published by Tobaldini et al. (2019b). The patients underwent ECG recordings and were neurologically assessed at the time of admission to the emergency department (T0), immediately after any reperfusion therapy (T1), 7 days (T7), and 3 months after the index incident. Patients with a major stroke at hospital admission (NIHSS score ≥14) presented with prevalent parasympathetic modulation. Moreover, the group with mRS 3–6 (after 3 months) presented a higher 2UV% (at the onset: 12.6 (8.8–20.2) vs. 20.7 (13–32.2), *p* = 0.04) and after 3 months: 11.8 (8.4–20) vs. 22.5 (13.6–31), *p* = 0.007) and lower 0V% (after 3 months (44.5 (29.6—58.4) vs. 26.6 (22.7—39), *p* = 0.032). Additionally, patients with right hemispheric lesions (n = 21) also presented a greater vagal modulation than those with left hemispheric lesions (n = 19), as shown by a greater 2LV% (5.7 (2.7–9.7) vs. 2.9 (1.2–4.4), *p* = 0.022). On the basis of these results, it was concluded that in the very early phases of AIS, a decreased 0V% and an increased 2UV% could reflect a loss of sympathetic oscillation, predicting unfavorable 3-month outcome after stroke. Tobaldini et al. (2019a) also assessed the autonomic control of the heart in different stages of sleep in patients with AIS. Using spectral, symbolic, and entropy analyses, they observed a loss of autonomic heart dynamics during sleep within the first 3 months after AIS or TIA. In ischemic stroke involving the insula, patients showed dominant vagal modulation and reduced sympathetic modulation during all sleep phases.

Chen et al. (2018), in addition to traditional methods of linear HRV analysis (SDNN, RMSSD, LF, HF, LF/HF ratio), used Multiscale Sample Entropy. The results indicate that a higher complexity of HRV (defined as the area under the Multiscale Sample Entropy curve) can predict a good functional outcome at 3 months post-stroke. The complexity index was lower in the ICH lobe groups (21.6 ± 7.9) than in the basal ganglia (27.9 ± 6.4) and thalamus (28.5 ± 7.2) groups.

#### 3.2.2 HRV metrics for prediction of stroke complications

##### 3.2.2.1 Mortality and recurrence of stroke

Recurrent stroke is a consequence of ischemic stroke and remains one of the greatest threats to life. The risk of relapse was 1.7%–4% in the 30 days after the first episode, 6%–12% in the first year, and 19%–42% in the next 5 years after the first stroke (Nowacki and Porębska, 2005). Guan et al. (2019), in a 2019 article, analyzed the 24-h Holter records of 201 patients (161 minor strokes; 40 TIA) within 48 h of the ischemic episode and followed the patients for 90 days. The risk of recurrence was evaluated using the ABCD2 score (age, BP, clinical features, duration of symptoms, and diabetes) (Johnston et al., 2007). The morning HF HRV and the changes in HF HRV from morning to afternoon were the best predictors of ischemic events (area under the curve [AUC] = 0.61 and 0.70) or ischemic stroke (AUC = 0.62 and 0.72). HRV-based models had a higher predictive value than the ABCD2 model for ischemic events (AUC = 0.82 vs. 0.63, 0.76 vs. 0.63; *p* < 0.05) and ischemic stroke (AUC = 0.87 vs. 0.64, 0.82 vs. 0.64, *p* < 0.05).

Chidambaram et al. (2017) analyzed 5-min ECGs to identify a link between autonomic dysfunction and morbidity and mortality associated with acute stroke. Almost 60% of the patients (60/97) had a higher LF/HF ratio, which was associated with a higher mortality. They also had significantly higher mean systolic BP, diastolic BP, and NIHSS than people with a reduced LF/HF ratio (166.33 ± 24.81 vs. 148.54 ± 19.42, *p* = 0.0003; 100.33 ± 18, 73 vs. 88.76 ± 12.66, *p* = 0.0013; and 15.77 ± 8.22 vs. 11.49 ± 6.63, *p* = 0.0088; respectively).

He et al. (2018) investigated whether percutaneous mastoid electrical stimulator could ameliorate abnormal HRV and reduce mortality in stroke survivors. FD evaluation 2 weeks after the application of percutaneous mastoid electrical stimulator indicated that the patients had a lower FD than that of the sham group (1.14 ± 0.27 vs. 1.00 ± 0.23; *p* = 0.001). In 2019, He et al. (2019), using FD, indicated the association between decreased HRV and early neurological deterioration and 1-year risk of recurrent ischemic stroke. FD ≤ 1.05 and FD ≤ 1.15 were independently associated with an increased risk of early neurological deterioration and recurrent ischemic strokes, respectively.

You et al. (2021) analyzed heart rate trajectories and variability (mean and coefficient of variation) as markers of functional outcome and death. The published results indicate that a high heart rate and greater variability in the acute phase of ICH are correlated with a higher risk of an unfavorable functional outcome (odds ratio 15.06, 95% CI 3.67–61.78).

Sykora et al. (2020) investigated the mortality of patients after ICH using non-linear HRV analysis. They showed that sample entropy, compared with several autonomous markers, provides the greatest amount of information about the likelihood of death after ICH.

A study by von Rennenberg et al. (2021) states the opposite of the above conclusions. They analyzed SDNN, RMSSD LF, HF, and LF/HF ratio in patients with AIS. Observations were made in the acute phase of stroke and 3 months and 1 year after the stroke. No association between HRV and mortality, recurrent stroke, myocardial infarction, or functional outcome was observed.

##### 3.2.2.2 Infections

Infections are a frequent complication of AIS, affecting 20%–35% of patients and adversely influencing survival and recovery (Elkind et al., 2020). The most critical are post-stroke pneumonia (12.3%) and urinary tract infections (7.9%) (Westendorp et al., 2011; Badve et al., 2019; de Jonge et al., 2020).

The study performed by Brämer et al. (2019) evaluated the predictive role of HRV metrics (mHR, TP, VLF, LFnorm, LF/HF, SDNN, and RMSSD) based on the 24-h Holter ECG performed in 287 patients within the first day of AIS onset. Clinical signs of infection, neurological status, and laboratory data were collected between days 1 and 5. HRV metrics were analyzed using a logistic regression model and the AUC. HRV was significantly associated with incident infection even after adjusting for clinical covariates (diabetes and NIHSS score at admission). A VLF band power was adjusted for predicting infection with AUC = 0.80 (cross-validation AUC = 0.74). A model with clinical data (diabetes, NIHSS, and involvement of the insular cortex) yielded similar results (AUC = 0.78, cross-validation AUC = 0.71). The authors commented that VLF HRV, an index of integrative autonomic-humoral control, could be considered as a biomarker of infection in the immediate post-stroke period.

Swor et al. (2019) analyzed the association between HRV and development of fever in patients with ICH. Lower SDNN occurred in patients with fever (SDNN: 1.72 vs. 2.55, *p* = 0.001). Lower HRV was positively correlated with persisting fever for greater number of days (R = −0.22, *p* < 0.001).

HRV spectral analysis by Wirtz et al. (2020) showed that in patients who developed nosocomial infection in the acute phase of stroke, the parasympathetic system was dominant. These patients had a higher normalized HF than in those without infection (0.54 vs. 0.36, *p* = 0.033), while the normalized LF in patients who developed a nosocomial infection on admission to the hospital was lower than that in those without infection (0.46 vs. 0.64, *p* = 0.033).

##### 3.2.2.3 Neurocardiogenic injury

Megjhani et al. (2020) analyzed 5-min ECG recordings of 326 patients with SAH. Fifty-six patients developed neurocardiogenic injury within the first 3 days after stroke. Researchers have observed that linear methods of HRV analysis can predict the development of neurocardiogenic injury during the acute phase of SAH. All measures of linear HRV analysis over the time domain (e.g., SDNN and RMSSD) decreased in patients with SAH and neurocardiogenic injury. Researchers also developed a machine learning classifier that, based on the classic HRV assessment measures, obtained results for the area under the receiver operating characteristic curve equal to 0.82, the area under the precision recall curve (0.75), and the correct classification coefficient of 0.81.

##### 3.2.2.4 Delirium

Studies performed in stroke units have shown that the incidence of delirium in patients with AIS is approximately 30% (Rollo et al., 2022). Moreover, delirium was an independent predictor of mortality during the 12 months period following hospital admission. Rollo et al. (2022) enrolled 56 patients (32 men and 24 women; mean age was 69.9 ± 13.3 years) with diagnosis of stroke with onset within the previous 72 h. Delirium was evaluated by the Richmond Agitation Sedation Scale and the Confusion Assessment Method-Intensive Care Unit at baseline and 72 h after admission. At the time of enrolment, no patient had clinical signs or Confusion Assessment Method-Intensive Care Unit scores consistent with delirium diagnosis. After 7 days, 11 patients developed delirium, and the study cohort was divided into two subgroups: patients with delirium (DLR+, 7 men and 4 women; mean age 69.9 ± 14.1 years) and patients without delirium (DLR-, 32 men and 13 women; mean age 69.9 ± 13.2 years). Patients in the delirium group had a smaller normalized HF (DLR: 38.23 ± 19.23 n.u.; DLR+: 25.75 ± 8.77 n.u.; *U*-test: 143.0; *p* = 0.031). No other significant differences were observed between the groups.

##### 3.2.2.5 Post-stroke depression

Post-stroke depression (PSD) is common, affecting approximately one-third of stroke survivors at any time after stroke (Medeiros et al., 2020). It is associated with higher mortality, poorer recovery, more pronounced cognitive deficits, and lower quality of life. Recently, He et al. (2020) investigated whether decreased HRV metrics were predictors of PSD in 503 consecutive patients with AIS who underwent 15 min 12-lead ECG monitoring and were screened for depression using the Hamilton Depression Rating Scale at baseline (within 7 days) and after 3 months. The autonomic function was assessed using FD. Receiver operating characteristic curve analysis revealed that the cut-off point for FD for early onset depression was ≤1.27 and ≤1.19 for 3-month depression. Moreover, patients with more severe stroke (adjusted OR, 1.15; 95% CI, 1.05–1.27; *p* = 0.005), younger age (adjusted OR, 1.18; 95% CI, 1.01–1.13; *p* = 0.046), and measured FD of ≤1.27 (adjusted OR, 3.31; 95% CI, 2.23–4.92; *p* < 0.001) had a significantly higher risk of early onset PSD. An increased incidence of depression at the 3-month follow-up was associated with a higher baseline NIHSS (adjusted OR, 1.21; 95% CI, 1.09–1.43; *p* < 0.001) and an FD of ≤1.19 (adjusted OR, 2.68; 95% CI, 1.79–4.03; *p* = 0.000). The authors concluded that lower HRV metrics and FD could be useful as potential predictors of PSD in patients with AIS.

In addition, Tessier et al. (2017) reported that a higher heart rate, lower HRV, and higher LF/HF ratio denoting sympathovagal balance were associated with a higher severity of depressive symptoms within the first week after stroke. Additionally, a longer observation period (3 months) showed that a higher sympathetic balance in the early phase was positively correlated with a greater intensity of depression symptoms in the future.

##### 3.2.2.6 Atrial fibrillation

Atrial fibrillation (AF) is an important stroke risk factor and is characterized by a marked HRV, which is possibly related to vagal tone; however, due to methodological issues, AF has rarely been evaluated in the context of HRV analyses (Khan et al., 2021). AF is one of the risk factors for stroke in the future; however, studies have described the risk of AF as a consequence of stroke. An attempt to predict the occurrence of paroxysmal AF episodes detected during hospitalization of patients with stroke was made by Adami et al. (2019). The study was conducted in a group of 200 patients. Analysis of RR interval fluctuations in the first hours after AIS identified 111 patients as having a low short-term risk of paroxysmal AF, 52 patients as high risk, and 37 patients with overt AF. Adami et al. stated that the dynamic analysis of RR performed in the acute phase of stroke indicates that patients are at low or high risk of developing paroxysmal AF episodes during hospitalization.

#### 3.2.3 Effects of heart rate variability biofeedback in patients with acute ischemic stroke

A new perspective on the role of HRV in patients after AIS was proposed by Chang et al. (2020). This study included a total of 35 patients. The main aim of this study was to investigate the effects of an HRV biofeedback (HRVBF) intervention on autonomic function, cognitive impairment, and psychological distress in patients with AIS. HRVBF is a behavioral intervention that allows patients to gain conscious control of higher HRV amplitudes. By stimulating breathing at a slow frequency so that resonance occurs between the cardiac rhythm and respiration, patients can enhance their HRV rates. The HRVBF group received four BF training sessions over 4 days, and the control group received the usual care. Three-way ECG recordings were performed at baseline, after 1 month, and after 3 months. Cognitive impairment was assessed using the Mini-Mental State Examination. For the HRVBF group, all time- and frequency-domain HRV parameters increased significantly at the 1- and 3-month follow-ups (*p* < 0.01). In addition, the HRVBF group showed significantly greater increases in SDNN (*p* = 0.005) and rMSSD (*p* = 0.01) than in the control group after 3 months. For the frequency-domain parameters, the intervention group had significantly greater increases than the control group at both 1 month (LF, *p* = 0.007; TP, *p* = 0.01) and 3 months (LF, *p* = 0.03; TP, *p* = 0.02).

Similar studies have been performed by Siepmann et al. (2021). They assessed the benefits of using HRVBF during the acute phase of stroke. For this purpose, they divided the sample study in two groups: the first group had a biofeedback session within the first 3 days of the onset of the stroke, and the second group (control) underwent sham biofeedback. After 3 months, follow-up was performed. HRV analysis parameters such as SDNN and RMSSD were assessed. The severity of autonomic symptoms was assessed at the beginning and after 3 months *via* a survey of the autonomic symptom scale Total Impact Score (TIS). The results demonstrate the effectiveness of using HRVBF sessions to improve neurocardiological function and permanently alleviate autonomic symptoms after AIS (within 3 months of stroke). Increase in HRV after biofeedback treatment (SDNN: 43.5 (79.0) ms at 3 months vs. 34.1 (45.0) ms on baseline, *p* = 0.015; RMSSD: 46.0 (140.6) ms at 3 months vs. 29.1 (52.2) ms at baseline, *p* = 0.015) was noted. Changes in TIS showed improvement in autonomic symptoms after 3 months (TIS 3.5 (8.0) at 3 months vs. 7.5 (7.0) at baseline, *p* = 0.029). In the control group subjected to a sham biofeedback session, no differences were observed in the SDNN, RMSSD, and TIS (SDNN, *p* = 0.63; RMSSD, *p* = 0.65; TIS, 0.006).

#### 3.2.4 HRV as a marker of stroke location

Aftyka et al. (2022) conducted a comprehensive analysis of the linear and non-linear parameters of HRV. They compared the patients in the acute phase of AIS. The results indicated that HRV may be a non-invasive test for detecting the location of a stroke in the cerebral brain (right or left hemisphere). The sample entropy results indicated that patients with left hemispheric stroke had significantly greater values than those with a right hemispheric stroke (1.31 ± 0.53 vs. 0.92 ± 0.46, *p* = 0.003). Patients with right hemispheric stroke had less HRV complexity than those with left hemisphere stroke.

## 4 Discussion

To the best of our knowledge, there is no current literature review that analyzes HRV as a predictor of stroke course, functional outcome, and medical complications in patients with stroke. This manuscript is an attempt to fill this gap and show the progress that has been made since 2018 (last published literature review by Lees et al. (2018)) with respect to correlation between HRV and stroke. The goal of this systematic review was to carry out an analysis of studies concerning the link between autonomic system activity measured by HRV and AIS outcomes. Although, in recent years, age-adjusted stroke incidence and mortality rates have tended to decrease, the prevalence is still increasing as a result of an aging population. Age and baseline stroke severity are well-established predictors of stroke survival; however, other prognostic factors are not well known. The clinical prediction of stroke recovery could be useful for clinical decision support and help in the early planning of rehabilitation and long-term management (Douiri et al., 2017). The extent of recovery varies among patients with stroke and strongly depends on lesion type and size. Both chronic risk factors and acute triggers of ischemic stroke are sources of stress to the body and are tightly associated with autonomic nervous system dysfunction, what can be assessed with HRV impairment (Guan et al., 2018). Identifying accurate prognostic and stress-related factors, especially modifiable ones, could be therefore important for clinicians, patients, and caregivers in the selection of appropriate treatment modalities, and also in providing information about expected outcomes. Changes in the cardiovascular system due to neurological disorders, such as stroke, are defined as the brainheart axis. Strokes, particularly those involving the insular lobe, are frequently associated with bradycardia, atrioventricular blocks, and tachyarrhythmias of either atrial or ventricular origin. These changes are among the most commonly observed alterations in the heart-brain axis and are probably associated with increased mortality in the acute phase of stroke, however, these effects could also be modulated by concomitant factors such as pre-existing cardiac diseases or electrolyte disorders. The results of the current review indicate that HRV in patients in the acute phase of stroke may serve as an important prognostic factor for the course and outcomes of stroke.

However, to date, the relationship between HRV and stroke outcomes is not fully understood.

Although easy access to HRV measurements has led to a number of studies assessing autonomic regulation in patients with stroke, significant caveats exist because of the complicated nature of HRV. First, most studies examined HRV in patients in the subacute (>7 days following stroke onset) or chronic phase of stroke (>6 months), whereas the most severe stroke complications, including autonomic dysfunction, appear in the acute phase of stroke. Second, most studies included only patients with minor stroke with less disability and smaller infarcts with frequent favorable outcomes and a low risk of cardiac complications, which increases proportionally to the severity of ischemic stroke and neurological deficits (Yoshimura et al., 2008; Guan et al., 2019; Zhang et al., 2019; Li et al., 2021). Third, the studies had different follow-ups, and the risk of stroke complications was the highest in the first 3 months after stroke. It has been reported that >60% of patients with AIS had ECG abnormalities of ischemic and/or arrhythmic character in the first 24 h after stroke onset, 19% of patients suffered from at least one serious cardiac adverse event during the first 3 months following stroke, and approximately 88% of patients with a stroke involving the insular cortex in the right cerebral hemisphere developed myocardial injury in the weeks following ischemic stroke (Lavy et al., 1974; Ay et al., 2006; Prosser et al., 2007).

The present systematic review provides evidence that HRV may be a useful predictor of acute and post-acute stroke outcomes. Different methodological aspects should be considered to properly derive HRV parameters and determine their clinical interpretations. Both the parasympathetic and sympathetic nervous systems are considered to be the main determinants of HRV-derived parameters in the time and frequency domains. A number of measures have been comprehensively analyzed by researchers and have shown promising prognostic value for different stroke outcomes. The majority of researchers have analyzed linear methods, the most common of which are frequency domain analysis and distinguishing of the parameters of spectrum power in the LF, HF, and their ratio (LF/HF).

Non-linear analyses, such as the observations of Poincare charts, could probably better describe the complex non-linear interactions between the parasympathetic and sympathetic nervous system branches and the behavior of the variations of consecutive R-R intervals; however, they have only occasionally been selected by researchers. Understanding entropy and fractal parameters is more difficult than understanding either time or frequency domain parameters; their physiological and clinical implications also remain unclear (Sassi et al., 2015). In recent years, in addition to the analysis of HRV with linear methods, non-linear HRV analysis has become more popular, particularly, entropy measures such as sample entropy (Aftyka et al., 2022; Castro et al., 2022). It is possible to infer the complexity of HRV by determining the entropy of the system. Another non-linear measure characterizing HRV is symbolic analysis (Tobaldini et al., 2019a; Tobaldini et al., 2019b).

HRV analysis parameters can be grouped into 3 categories: linear methods in the time domain (e.g., meanNN, SDNN, RMSSD, pNN50), linear methods in the frequency domain (e.g., HF, LF, VLF, and ULF) and non-linear methods (as symbolic dynamics, entropy, fractal dimension etc.). The first 2 categories—linear methods in the time and frequency domain are definitely mathematically simpler, easier to understand than non-linear methods. On the other hand, heart rate variability is characterized by non-linearity, therefore non-linear signal analysis methods should be used, because some HRV characteristics may be invisible after using linear methods, and will become visible only during the analysis using non-linear methods. Signal duration is crucial when choosing an HRV analysis method. The gold standard is 24-h recordings, however, we cannot always obtain such a long signal in the case of short-term recording, not all methods of HRV analysis can be used. As indicated by Shaffer and Ginsberg (Shaffer and Ginsberg, 2017) e.g., SDNN 24 h predicts future heart attack risk and the shorter 5-min record is not. Similarly, the power spectrum in ultra low frequency (ULF) should be determined for 24 h recording. It is impossible to clearly indicate which parameters of the HRV analysis should be used, because some parameters complement each other. Task Force of the European Society of Cardiology (Guidelines of the Task Force of the European Society of Cardiology the North American Society of Pacing Electrophysiology, 1996) indicate that both linear methods in the time domain (such as: meanNN, SDNN, RMSSD, pNN50) and linear methods in the frequency domain (in particular HF, LF, VLF) should be analyzed. Non-linear methods currently do not belong to the gold standard commonly used in assessing HRV, as they are new but very promising measures.

The current study has several limitations. We attempted to control the research methodology to the fullest extent; however, the most important limitation was the heterogeneity of the population, measures and the different timing of the HRV assessment, which did not allow us to perform a meta-analysis that would have been more conclusive. Therefore, it is necessary to better understand whether some HRV-derived parameters are more reproducible than others are. The strategy to include only peer-reviewed journals published in English Language may have limited the selection of studies that have provided results in line with the literature, and they could have overestimated this relationship and led to the elimination of research conducted on other populations, further limiting the generalizability of the results. Another limitation is that, in this manuscript, we also analyzed studies that did not exclude people with AF (cardiac condition that affects HRV). As a result, the HRV scores of people after a flicker stroke vs. those without AF can be considered equal. However, we decided to exclude articles in which people with AF were not excluded, as this factor was present in several of the analyzed studies. In addition, a large proportion of stroke survivors have developed AF, which is one of the factors contributing to stroke onset.

This review concludes that HRV could be considered a promising biomarker for stroke outcomes and complications. However, researchers and clinicians should consider several methodological aspects to appropriately quantify and interpret the HRV-derived parameters. These results highlight the influence of the autonomic nervous system on both short- and long-term stroke outcomes.

## Data Availability

The original contributions presented in the study are included in the article/Supplementary Material, further inquiries can be directed to the corresponding author.
