# Supplementary material for: Heart rate variability as a predictor of stroke course, functional outcome, and medical complications: A systematic review
Source: Front Physiol. 2023 Feb 9;14:1115164. doi: 10.3389/fphys.2023.1115164 (PMC9947292; doi:10.3389/fphys.2023.1115164)
Supplement: Supplementary file 1 [file DataSheet1.PDF]

Supplementary Material

1 Table

Table 1: Overview of the included articles. Main data collected from each of the 36 included articles.

Author, Year	Study population	Objective	Time from stroke onset to HRV measurement	Method of HRV analysis	HRV parameters	Endpoint	Follow-up	Study design
Sethi et al., 2016	13 patients with AIS (6 women; 7 men; mean age 61 ± 12 years)	association beetween HRV and motor outcome	within 3 days from hospital admission	24 h Holter monitor	SDNN	motor outcome after stroke	3 months	cohort study
Xu et al., 2016	63 patients with AIS (25 women; 38 men; mean age 71 ± 12 years); 50 controls with high risk of stroke	investigate the cardiac autonomic function of patients with AIS	within 72 hours after stroke	24 h Holter ECG	SDNN, RMSSD	autonomic dysfunction	24 h	cohort study

Chidambaram et al., 2017	97 patients with AIS (41 women; 56 men; mean age 60.84 ± 14.12 years)	investigation the pattern of autonomic dysfunction among patients admitted to acute stroke and the relationship between autonomic dysfunction and morbidity and mortality associated with acute stroke	less than 7 days prior to admission	5 min resting ECG	HF, LF, HF/LF	autonomic dysfunction, morbidity, mortality associated with acute stroke	30 days	cohort study
Tessier et al., 2017	54 patients with AIS (17 women; 37 men; mean age 51.7 ± 13 years)	association between HRV and early-phase of depression and cognition	2–7 days after admission	30 min morning intervals (8:00-8:30)	mean heart rate, RMSSD, LF, HF, LF/HF	cognitive functions	3 months	cohort study

Wei et al., 2017	232 patients with AIS (98 women; 134 men; mean age 69 ± 19 years)	association between autonomic function and stroke in patients with renal dysfunction	2–7 days after admission	24 h Holter ECG	SDNN, SDNN index, SDANN index, RMSSD, pNN50, TP, VLF, LF, HF, LF/HF	autonomic dysfunction	24 h	cross-sectional observational study
Chen et al., 2018	93 patients with ICH (38 women; 55 men; mean age 61.1 ± 15.3 years); 50 controls (60% male; age 60.2 ± 10.8 years)	influence of ICH and IVH locations on the complexity of HRV	within 24 h from hospital admission	1 h ECG	SDNN, RMSSD, HF, LF, HF/LF, MSE	functional outcome	3 months	cohort study
He et al., 2018	130 patients with AIS (60 women; 70 men;	alleviation abnormal HRV using percutaneous mastoid	within 3 days of symptom onset	short-time: 512 RR intervals	fractal dimension (FD)	death, major disability	2 weeks, 3 months	prospective, randomized, double-blinded, placeb

	mean age 68.00 ± 10.30 years)	electrical stimulator (PMES)						o-controlled study
Szabo et al., 2018	47 patients with ICH (12 women; 26 men; mean age 63.2 ± 8.7 years) and 47 controls (7 women; 11 men; mean age 59.6 ± 7.8 years)	associations between HRV and stroke severity, hemorrhage volume and outcome after ICH	first 24 h after onset of symptoms	short-time (300s)	HF, LF, HF/LF	autonomic changes, functional outcome and mortality	24 h, 3 months	cohort study
Adami et al., 2019	200 patients: AIS 187, TIA 13 (80 women; 120 men; mean age 76 ± 16 years)	an attempt to identify patients with acute ischemic stroke at a higher risk of subsequ	the first 48h after stroke	24 h Holter	RR fluctuation; Poincare plots	another episode of paroxysmal atrial fibrillation	48 h	cohort study

		ent episodes of paroxysmal atrial fibrillation detected during hospitalization due to stroke based on the analysis of RR in the first hours after admission to the ward						
Bramer et al., 2019	89 patients with AIS (39 women; 50 men; mean age 65 years)	predictive value of HRV in identifying increased risk of infection, SIRS or severe sepsis after AIS	first day after stroke onset	24 h Holter ECG recordings	mHR, TP, VLF, LF/HF, SDNN, RMSSD	an incident of infection occurring during the post-acute interval from days 3 to 5 after stroke onset	5 days	cohort study
Brunetti et al., 2019	42 patients: 21	examining the autono	polysomnography within 3	full polysomnography night	LF, HF, LH/H	autonomic dysfunction in acute	3 days	cohort study

	LH and 21 RH AIS and 42 controls (20 women; 22 men; mean age 69.8 ± 11.3 years)	mic modifications of wakefulness and sleep in a cohort of patients with acute ischemic stroke and assessing whether these modifications were dependent on the sleep phase and laterality of the stroke	days of stroke onset	recording	F, meanRR	ischemic stroke		
Guan et al., 2019	201 patients: 161 minor stroke, 40 TIA (38 women; 163 men; mean age 59 ± 10	role of HRV in prediction on the occurrence of secondary ischemic events after TIA or minor stroke	48h	24 h Holter	HF	occurrence of AIS, TIA, cardiovascular events and vascular death within 90 days after the initial TIA or minor stroke	90 days	cohort study

	years)							
He et al., 2019	516 patients with RIS (263 women; 253 men; mean age 66.14 ± 10.11 years)	relationship between HRV and early neurological deterioration	within 72 h from symptom onset	the portable 12-lead ECG 15 minutes examination	fractal dimension (FD)	association between decreased HRV and early neurological deterioration and 1-year risk of recurrent ischemic stroke	every month for 1 year	cohort study
Swor et al., 2019	248 patients with ICH [125 women; 123 men; mean age 63 (IQR 54-74) years]	association between HRV and development of fever in patients with ICH	within 24 h of admission to the hospital	10 second ECG	SDNN, RMSSD	fever occurrence	14 days	cohort study
Tian et al., 2019	53 patients with AIS (8 women; 45 men; mean age 65.34 years); 13 controls ≥45	association between HRV and autonomic dysregulation	within 7 days after onset	120-s recordings of RR interval	VLF, LF, HF, LF/HF, TP	autonomic dysregulation	7 days	cohort study

	years							
Tsai et al., 2019	34 patients with AIS (12 women; 26 men; mean age 63.2 ± 8.7 years) and 18 controls (7 women; 11 men; mean age 59.6 ± 7.8 years)	differences in baroreflex sensitivity depending on the subtypes of AIS	24h after stroke	2h ECG	HF, LF, VLF	baroreflex sensitivity	7 days; 3 months	cohort study
Tobaldini et al., 2019a	41 patients with AIS: 27 RT (13 women; 28 men; mean age 68 ± 12.8 years)	prognostic value of HRV and the role of stroke localization and RT on autonomic control	at the time of admission to the emergency department	thoracic piezoelectric belt with an ad hoc telemetric system device for 10 minutes	VLF, LF, HF, LF/HF; symbolic analysis: 0V %, 2LV% and 2UV%	neurological outcome	3 months	cohort study
Tobaldini et al., 2019b	45 patients with AIS [14	cardiac autonomic control in	within 7 days from onset of symptoms	polysomnographic recordings; 6-lead ECG	LF, HF, LF/HF, symbo	dysfunction of cardiac autonomic dynamics during sleep	3 months	cohort study

	women; 31 men; mean age 56 (range 30-74) years]	different sleep stages			lic analysis (0V %, 1V %, 2LV %, 2UV %), corrected conditional entropy			
Candemir and Onder, 2020	148 (74 RH and 74 LH) patients with AIS and 80 control subjects (63 women; 85 men; mean age 66.72 ± 13.07 years)	difference between HRV and HRT; differences in the involvement of the ANS between RH and LH strokes	during first week after AIS	Holter ECG 20 h monitoring	SDSD, SDNN, SDANN, RMSSD, pNN50, VLF, LF, HF, LF/HF	cardiac mortality and morbidity after stroke	1 week	cross-sectional study
Chang et al., 2020	35 patients: 19 AIS, 16 controls (17 wome	effects of an HRVBF intervention on autonomic function	at baseline and after 1 month and 3 months	three-way ECG	SDNN, RMSSD, VLF, LF, HF	cognitive impairment, psychological distress	3 months	randomized controlled trial

	n; 18 men; mean age 67.4 ± 9.7 years)	, cognitive impairment and psychological distress						
He et al., 2020	503 patients with AIS (257 women; 246 men; mean age 65.93 ± 10.19 years)	association beetween HRV and poststroke depression	within 72 h from symptom onset	1 h ECG	fractal dimension (FD)	occurrence of post stroke depression	3 months	cohort study
Megjhani et al., 2020	326 patients with SAH: 56 (17.18 %) subjects had NCI; (227 women; 99 men; mean age 55.70 ± 14.06 years)	relationship between HRV and NCI after SAH detection	within 48 after symptoms onset	300-s (5-min) epochs of ECG data	R-R intervals, SDNN, RMSSD, SD1, SD2, and their ratio, LF, HF LF/HF ratio.	NCI developing	3 days	cohort study
Sykora	47	associat	first 24 h	short-time	Sampl	mortality	3	cohort

et al., 2020	patients with ICH (20 women; 27 men; mean age 60.8 ± 16.5 years)	ion of sample heart rate entropy with mortality after ICH	after stroke onset	(300s)	e Entropy, VLF, LF, HF, LF/HF, TP		months	study
Tang et al., 2020	142 patients with AIS (17 women; 125 men; mean age 63.9 ± 10.2 years)	prediction of the functional outcome of a stroke based on HRV	within 7 days from an ischemic stroke	5 min ECG	TP, VLF, LF, HF, LF/HF	functional outcome	3 months	cohort study
Wirtz et al., 2020	64 patients: 9 - ischemic stroke; 5 - hemorrhagic stroke; 50 - TBI (with or without subsequent	correlation between ANS activity and immune response	at admission and during their ICU or medium care stay	10-min recording at admission and 10-min recording four times daily during the admission period	LF_{nu}, HF_{nu}, LF/HF	nosocomial infections and immunosuppression, mortality	28 days	cohort study

	hemorrhagic stroke) (17 women; 47 men; mean age 51 ± 19.3 years)							
Zhao et al., 2020	186 patients AIS (36 women; 150 men; mean age 60 years)	association between HRV and infarct locations, etiology sub-types, and neurological functional outcomes in patients with AIS	3-7 days after admission	24 h ECG	SDNN, mRR, RMSSD, pNN50	autonomic dysfunction	3 months; 6 months; 1 year after stroke	prospective observational study
Li et al., 2021	5308 patients with minor stroke or TIA (1640 women; 3668 men; mean	correlation between HRV and 90-day outcomes	during hospitalization	24 h ECG	SDNN, RMSSD	neurological disability, stroke recurrence and cardiovascular death during 90 days after stroke	follow-up was conducted at 3 months, and telephone follo	prospective registration study

	age 61.13 ± 10.81 years)						w-up was conducted at 6 months and 1–5 years	
Miwa et al., 2021	994 patients with ICH [379 women; 615 men; mean age 62 (IQR 52-71) years]	impact of HR and HRV on poorer clinical outcomes	first 24 h after ICH	maximum and minimum HR for every 15 minutes for the first hour and every hour thereafter between 1 and 24 hours of hospitalization	mean HR, std HR, HR-CV, HR-SV, HR-AVR	clinical outcome	3 months	cohort study
Siepmann et al., 2021	48 patients with AIS who received nine 10-min sessions of HRV biofeedback (19 women; 29 men; mean	influence HRV biofeedback on improves neurocardiac function by modulating ANS activity after AIS	during hospitalization at stroke unit care	3 min ECG	SDNN, RMSSD	neurocardiac dysfunction worsens clinical outcome and increases mortality in stroke	3 months	randomized sham-controlled pilot study

	age 66 ± 4.4 years)							
von Rennenberg et al., 2021	308 patients with AIS [117 women; 191 men; mean age 69 (IQR 58-75) years]	association between HRV and mortality, recurrent stroke, myocardial infarction or functional outcome	median daytime 57 h (95% CI 38–82 h); median nighttime 67 h (95% CI 47–92 h)	10 min ECG (daytime (6 p.m. ± 1 h) & nighttime (3 a.m. ± 1 h)	SDNN, RMSSD, LF, HF, LF/HF	mortality, recurrent stroke, myocardial infarction or functional outcome	telephone interviews 3 and 12 months after stroke	cross-sectional study
You et al., 2021	332 patients with ICH (108 women; 224 men; mean age 64.3 ± 13.7 years)	associations of heart rate trajectories and variability with functional outcome and mortality	first 72 h of hospitalization	at baseline, every 6 h in the first 24 h after enrollment, and at 48 and 72 h	heart rate trajectories and variability (mean and coefficient of variation)	functional outcome, death	3 months	prospective study
Zhang et al., 2021	106 patients with ELVO (48 women; 58 men; mean	correlation between HRV and long-term outcome in	during MT	ECG monitoring during MT	LF, HF, LF/HF	functional outcome	90 days	retrospective study

	age 62.7 ± 8.2 years)	patients who received MT for ELVO						
Aftyka et al., 2022	64 patients with AIS: LH: 20 women; 19 men; mean age 66 ± 13 years RH:12 women; 13 men; mean age 64 ± 12 years	relationship between HRV and the hemisphere of the stroke	at a mean of 4.3 ± 2 days following AIS	24-hours ECG	mean NN, SDNN, RMSSD, pNN50, LF, HF, LF/HF ratio, Sample Entropy	hemispheric involvement in AIS, functional outcome	30 days, 90 days, and 12 months	cohort study
Castro et al., 2022	26 patients with AIS in middle cerebral artery (10 women; 16 men; mean age 73 ± 12 years)	relationship between HRV and BRS in dynamic cerebral autoregulation in the early hours post ischemia, and its impact	within 6 hours (median time from stroke onset was 285 min)	short time ECG	SDNN, LF, HF, LF/HF, LF normalized, HF normalized , Sample Entropy, Fuzzy Entropy, baroreflex	increased vagal modulation in early hours of AIS, may interfere with cerebrovascular control and is associated with larger infarcts	at discharge, 7 days and 90 days	cohort study

		in clinical and radiological outcome			sensitivity, blood pressure variability			
Rollo et al., 2022	56 patients with AIS (24 women; 32 men; mean age 69.9 ± 13.2 years)	relationship between autonomic activity measuring by HRV analysis and predisposition to delirium	within 24 h from admission	short time ECG	mean HR, STD, meanRR, SDNN, RMSSD, NN50, pNN50, triangular index, TINN, SD1, SD2	acute non-cardioembolic stroke patients with increased variability of heart rate and decreased vagal control are at risk for delirium	7 days	prospective, cross-sectional, cohort study
Wang et al., 2022	61 patients with PCIS (19 women; 42 men; mean age 66.5) and 30 control subjects (17 women; 13 men; mean	CAM assessment in patients with PCIS with and without brainstem involvement	during the first week after stroke onset	5 min records; 3-lead ECG; finger-pulse photoplethysmography; piezoelectric respiratory belt;	RR intervals, stdRR, RMSSD, SBP, DBP, TP, LF, HF, LF/HF	cardiovascular autonomic dysfunction	7 days	cohort study

	age 63.3 ± 6.8 years)							

RH – right hemisphere; LH- left hemisphere; HRV - heart rate variability; HRT – heart rate turbulence; AIS – acute ischemic stroke; ICH - intracerebral hemorrhage; SAH - subarachnoid hemorrhage; RIS- recurrent ischemic stroke; NCI - neurocardiogenic injury; TBI - traumatic brain injury; IVH - intraventricular hemorrhage; TIA – transient ischemic attack; ANS – autonomic nervus system; ICU – intensive care unit; ECG – electrocardiography; NN - normal-to-normal interval; SDSD - standard deviation of the normal-to-normal; SDNN - standard deviation of the interval NN; SDNN index - the mean of the standard deviations of the normal RR intervals in all 5-min segments in the entire 24-h recording; SDANN - the standard deviation of the average NN interval; SDANN index - the mean of the standard deviation of the averaged normal-to-normal RR intervals in all 5-min segments of the 24-h recording; RMSSD- the square root of the mean squared differences of successive NN intervals; pNN50 - the division of the number of interval differences of successive NN intervals of more than 50 ms by the total number of NN intervals; TP – total spectral power; VLF - very low frequency; LF - low frequency; HF - high frequency; LF/HF -low frequency/high-frequency ratio; MSE – Multiscale Sample Entropy; TO - % change RR intervals after VPBs compare to pre-VPB period; TS - the maximum positive regression slope obtained over any five consecutive sinus RR intervals within the first 15 sinus RR intervals following the VPB; VBP - ventricular premature beats; RT - reperfusion treatment; 0V% - pattern with no variation; 1V% - pattern with one variation; 2 LV% - pattern with two like variations; 2UV% - pattern with two unlike variations; ELVO- early large vessel occlusion; MT – mechanical trombectomy; SIRS - systemic inflammatory response syndrome; mHR – mean heart rate; mRR – mean RR interval; PSD – post stroke depression; FD- fractal dimension; HRVBF – HRV biofeedback; TINN – triangular interpolation of NN interval histogram; stdRR – standard deviation of RR intervals; PCIS - posterior circulation ischemic stroke; CAM - cardiovascular autonomic modulation; HR-CV – heart rate coefficient of variation; HR-SV – heart rate successive variation; HR-ARV – heart rate average real variability

Table 2: Study quality analysis by the Newcastle-Ottawa Scale.

Table 2. Quality of Included studies analised with Newcastle- Ottawa Quality Assessment Scale.

	**Selection**				**Comparability**		**Outcome/ Exposure***			**Results**				**Score**
Study (1st Author /year)	Representativeness of the exposed cohort	Selection of the non exposed cohort	Ascertainment of exposure	Demonstration that outcome of interest was not present at start of study	Comparability	Comparability for any additional factor	Assessment of outcome	Was follow-up long enough for outcomes to occur	Adequacy of follow up of cohorts	Selection	Comparability	Outcome/ Exposure	Total Score	high (7–9), medium (4–6), low (0–3)
Sethi et al., 2016		*	*					*	*	2	0	2	4	medium

Supplementary Material

Xu et al., 2016		*	*						*	2	1	1	4	medium
Chidambaram et al., 2017		*	*					*	*	2	0	2	4	medium
Tessier et al., 2017		*	*		*	*	*	*	*	2	2	3	7	high
Wei et al., 2017	*	*	*	*	*		*		*	4	1	2	7	high
Chen et al., 2018		*	*	*	*	*	*			4	2	1	7	high
He et al., 2018	*	*	*		*		*			3	1	1	5	medium
Szabo et al., 2018		*	*		*			*	*	2	1	2	5	medium
Adami et al., 2019		*	*		*		*			2	1	1	4	medium
Bramer et al., 2019	*	*	*		*		*			3	1	1	5	medium
Brunetti et al., 2019		*	*	*			*		*	3	0	2	5	medium

Guan et al., 2019		*	*				*	*		2	0	2	4	medium
He et al., 2019	*	*	*				*	*	*	3	0	3	6	medium
Swor et al., 2019	*	*	*				*		*	3	0	2	5	medium
Tian et al., 2019		*	*				*		*	2	0	2	4	medium
Tsai et al., 2019		*	*		*		*	*	*	2	1	3	6	medium
Tobaldini et al., 2019a		*	*	*	*		*	*	*	3	1	3	7	high
Tobaldini et al., 2019b		*	*	*			*	*	*	3	0	3	6	medium
Candemir and Onder, 2020	*		*	*			*			3	0	1	4	medium
Chang et al., 2020				*	*		*	*		1	1	2	4	medium

He et al., 2020	*	*	*				*	*	*	3	0	3	6	medium
Megjhani et al., 2020		*	*				*		*	2	0	2	4	medium
Sykora et al., 2020		*	*				*	*	*	2	0	3	5	medium
Tang et al., 2020		*	*				*	*	*	2	0	3	5	medium
Witz et al., 2020		*	*				*		*	2	0	2	4	medium
Zhao et al., 2020		*	*				*	*	*	2	0	3	5	medium
Li et al., 2021		*	*	*			*	*	*	3	0	3	6	medium
Miwa et al., 2021		*	*				*	*	*	2	0	3	5	medium
Siepman et al., 2021		*	*				*	*	*	2	0	3	5	medium
von Rennenberg et al., 2021		*	*					*	*	2	0	2	4	medium

You et al., 2021		*	*	*			*	*	*	3	0	3	6	medium
Zhang et al., 2021	*	*	*	*			*	*	*	4	0	3	7	high
Aftyka et al., 2022		*	*	*			*	*	*	3	0	3	6	medium
Castro et al, 2022		*	*	*	*		*		*	3	1	2	6	medium
Rollo et al., 2022		*	*				*		*	2	0	2	4	medium
Wang et al., 2022	*	*	*	*			*		*	4	0	2	6	medium
*Outcome for case control studies and Exposure for cohort studies														

Supplementary Material

Table 3: Association of HRV and AIS or ICH evaluated by non-linear methods.

Autror, Year	Non-linear methods results	Non-linear methods characteristic
Tobaldini et al. 2019b<br>Tobaldini et al. 2019a	• A higher 2UV% in patients with mRS 3-6 (at the onset: 12.6 (8.8–20.2) vs. 20.7 (13–32.2), p = 0.04) and after 3 months: 11.8 (8.4–20) vs. 22.5 (13.6–31), p = 0.007) and lower 0V% (after 3 months (44.5 (29.6 – 58.4) vs. 26.6 (22.7 – 39), p = 0.032).<br>• Patients with RH lesions (n=21) presented a greater 2LV% than those with LH lesions (n=19) (5.7 (2.7–9.7) vs. 2.9 (1.2–4.4), p = 0.022).<br>• A decreased 0V% and an increased 2UV% may predict unfavorable 3-month outcome after stroke.	• Symbolic dynamics is a new non-linear method, which may be used to detect non-reciprocal changes of sympathetic and parasympathetic modulation in healthy and diseased patients<br>• It is based on the transformation of time series into a sequence of symbols, the construction of patterns, the reduction of patterns into four families, and the evaluation of their occurrence.<br>• Four families can be identified: 0V%, pattern with no variation, 1V %, pattern with one variation, 2LV%, patterns with two like variations, and 2UV% pattern with two unlike variations.<br>• The 0V% group means the percentage share in the entire signal of such 3 intervals that are the same symbol, which means greater stationarity of the signal in a short window, and 2UV% describes such 3 adjacent intervals that have 3 different symbols, i.e. acceleration or deceleration of HRV in short time window.<br>• Using non-linear-symbolic dynamic methods, it is possible to present results that cannot be demonstrated by classical methods.
Chen et al. 2018<br>Sykora et al. 2020	• A higher area under the Multiscale Sample Entropy curve can predict a good functional outcome at 3 months post-stroke.<br>• The complexity index was lower in the ICH lobe groups (21.6 ± 7.9) than in the basal ganglia (27.9 ± 6.4) and thalamus (28.5 ± 7.2) groups.	• Multiscale Sample Entropy is a more extensive Sample Entropy method.<br>• This method refers to signal complexity. Sample entropy can detect some values that cannot be visualized in linear measures because entropy is less sensitive to single beats other than sinus rhythm.<br>• Non-linear methods of HRV analysis can be compared to a magnifying glass that gives a better look at the behavior of heart rate variability.
